# Increased Aortic Calpain-1 Activity Mediates Age-Associated Angiotensin II Signaling of Vascular Smooth Muscle Cells

**DOI:** 10.1371/journal.pone.0002231

**Published:** 2008-05-21

**Authors:** Liqun Jiang, Mingyi Wang, Jing Zhang, Robert E. Monticone, Richard Telljohann, Gaia Spinetti, Gianfranco Pintus, Edward G. Lakatta

**Affiliations:** Laboratory of Cardiovascular Science, National Institute on Aging, National Institutes of Health, Baltimore, Maryland, United States of America; Laboratorio di Patologia Vascolare, Istituto Dermopatico dell'Immacolata, Italy

## Abstract

**Background:**

Angiotensin II (Ang II) signaling, including matrix metalloproteinase type II (MMP2) activation, has been linked to an age-associated increase in migration capacity of vascular smooth muscle cells (VSMC), and to other proinflammatory features of arterial aging. Calpain-1 activation is required for MMP2 expression in fibroblasts and is induced in cardiomyocytes by Ang II. The consequences of engagement of calpain-1 with its substrates, however, in governing the age-associated proinflammatory status within the arterial wall, remains unknown.

**Methodology/Principal Findings:**

The present findings demonstrate that transcription, translation, and activity of calpain-1 are significantly up-regulated in rat aortae or early-passage aortic VSMC from old (30-mo) rats compared to young (8-mo). Dual immunolabeling of the arterial wall indicates that colocalization of calpain-1 and Ang II increases within the aged arterial wall. To further explore the relationship of calpain-1 to Ang II, we chronically infused Ang II into young rats, and treated cultured aortic rings or VSMC with Ang II. We also constructed adenoviruses harboring calpain-1 (CANP1) or its endogenous inhibitor calpastatin (CAST) and infected these into VSMC. Ang II induces calpain-1 expression in the aortic walls in vivo and ex vivo and VSMC in vitro. The Ang II mediated, age-associated increased MMP2 activity and migration in VSMC are both blocked by calpain inhibitor 1 or CAST. Over-expression of calpain-1 in young VSMC results in cleavage of intact vimentin, and an increased migratory capacity mimicking that of old VSMC, which is blocked by the MMP inhibitor, GM6001.

**Conclusions/Significance:**

Calpain-1 activation is a pivotal molecular event in the age-associated arterial Ang II/MMP2 signaling cascade that is linked to cytoskeleton protein restructuring, and VSMC migration. Therefore, targeting calpain-1 has the potential to delay or reverse the arterial remodeling that underlies age-associated diseases i.e. atherosclerosis.

## Introduction

A growing body of evidence indicates that aging is the major risk factor for the development of the quintessential cardiovascular diseases i.e. atherosclerosis, hypertension, and stroke [1]–[8]. It is well-known that aging induces several changes in vascular structure and function, i.e., increases in arterial intimal-medial thickness, arterial stiffness, and a proinflammatory state [1]–[8]. These alterations appear to be closely linked to enhanced arterial wall angiotensin II (Ang II) signaling [9]–[12]. Prior studies have demonstrated that the arterial wall or vascular smooth muscle cells (VSMC) of old rats have an over-abundance of the renin angiotensin system (RAS) components, including Ang II and its downstream molecular cascade, e.g. transforming growth factor-beta1 (TGF- β 1), extracellular matrix metalloproteinase type II (MMP2), endothelin-1 (ET-1), monocyte chemoattractant protein -1 (MCP-1), and reactive oxygen species (ROS) [8]–[17].

Calpain-1, a ubiquitous, cytosolic Ca^2+^ activated neutral protease, is a heterodimeric molecule which consists of a large 80 kDa catalytic and a small 30 kDa regulatory subunit [18], [19]. Calpain-1 activation is linked to the cellular Ca^2+^ loading status in a dose–dependent manner within living cells [20]. Prior studies indicate that calpain is of potential importance in the regulation of the proteolysis of key enzymes and structural proteins as well as proinflammatory responses [21]–[28]. Calpain-1 mediates MMP2 expression, and consequently affects the invasion of fibroblasts and leukemic cells [23], [24]. Increased MMP2/9 is a characteristic feature of central arterial aging in rats, nonhuman primates, and humans [8], [11], [12], [29], and MMP2/9 activation plays a crucial role in age-associated VSMC migration, elastin degradation, and collagen deposition [11], [30], [31]. In addition, the proteolysis of vimentin and spectrin by calpain-1 is required for the attachment and spreading of fibroblasts [18]–[22]; Further, calpain-1 activation is necessary for the cardiomyocyte hypertrophy induced by Ang II [32] and for the MCP-1 secretion from VSMC stimulated by IL-1α [25]. In fibroblasts calpain-1 is also an effective activator of the profibrogen TGF-β1, which results in the production of extracellular matrices [31], [33].

The consequences of the physiological engagement of calpain-1 with its substrates are important because the spectrum of calpain actions strikingly resemble those that accompany aging of the arterial wall. We hypothesized that calpain-1 activity increases with aging and mediates some of the aforementioned aspects of the age-associated arterial wall proinflammatory state. In the present study, we demonstrate that the levels of transcription, translation, and activation of calpain-1 are significantly up-regulated in rat aorta or early-passage aortic VSMC from old rats compared to their young counterparts. In an attempt to define a specific role for calpain-1 in the arterial aging process, we constructed recombinant adenoviruses harboring cDNA of calpain-1 or its endogenous inhibitor calpastatin, and infected VSMC. We also infused Ang II into rats to determine its effect on calpain-1 levels and activity. The results show that calpain-1 activation appears to be a pivotal event in the age-associated arterial Ang II/MMP2 signaling cascade, including MMP2 activation, cytoskeleton protein restructuring, and VSMC migration. Thus, calpain-1 activation is central to the initiation and progression of proinflammation in the arterial wall with advancing aging.

## Results

### The levels of calpain-1 transcript, protein abundance and activity increase within the aged aorta

**Figure 1:**
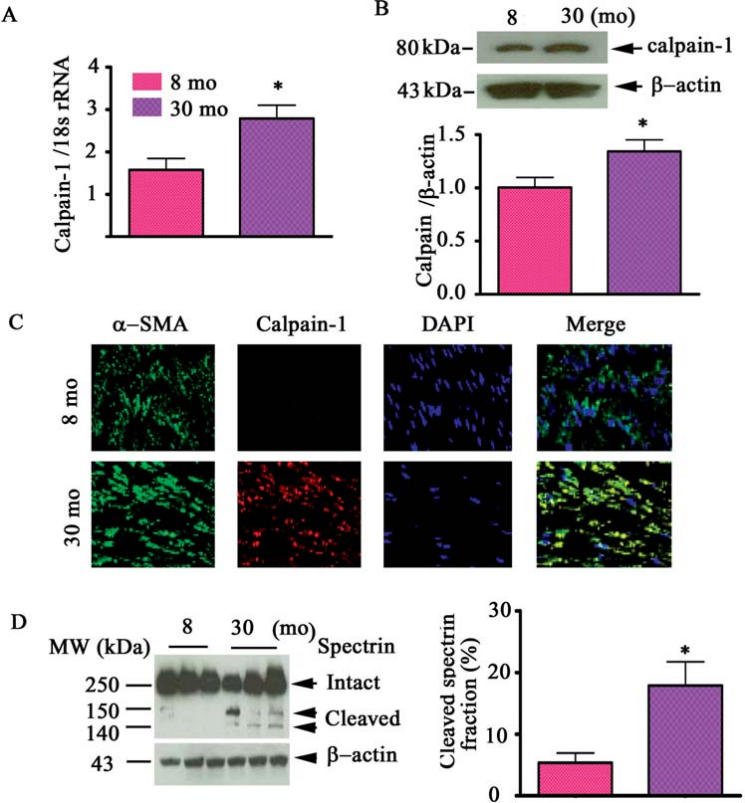
Calpain-1 transcriptome, protein abundance, and activity increase in the aged aortic wall. A. Average data of calpain-1 transcriptome. B. Western blots for calpain-1 protein of rat intact aortae (upper panel); average data (lower panel). C. Dual en face fluorescence staining for α-SMA (green), calpain-1 (red) in the medial aortic sections from young (upper panels) and old rats (lower panels). Nuclei were counterstained with DAPI (blue). Merged image is depicted in right panel (yellow-blue). Magnification: ×400. D. Western blots for calpain substrate α-II spectrin from aortic lysates (left panels). Average cleaved spectrin fraction (right panel). *p<0.05, old vs. young.

The quantitative reverse transcription polymerase chain reaction (qRT-PCR) showed that the transcript level of calpain-1 increased 1.8 fold within the aortic wall in old (30 mo) compared to young (8 mo) rats (**Fig. 1A**); Western blot analysis indicated that calpain-1 protein abundance increased 1.3-fold (p<0.05) with age (**Fig. 1B**). In addition, dual immunolabeling of *en face* medial aortic sections demonstrated that increased calpain-1 protein within old rats was co-localized with α-smooth muscle actin (α-SMA), a marker of VSMC, stained cells **(** **Fig. 1C**, lower right-most panel). The levels of cleaved spectrin fragments predict calpain activity. In aortic lysates of old rats **Fig. 1D** **(**left panel) three conspicuous spectrin bands, designated as intact (250 kDa) and cleaved spectrin fragments (145, and 150 kDa) respectively, are observed. In contrast, only intact spectrin was detected in young rat aortic lysates. Thus, on the basis of cleaved spectrin fragments old rats have a 3.3 fold increase in aortic calpain activity compared to their young counterparts (**Fig. 1D**, right panel).

### Ang II enhances calpain-1 transcription, translation, and activity

**Figure 2:**
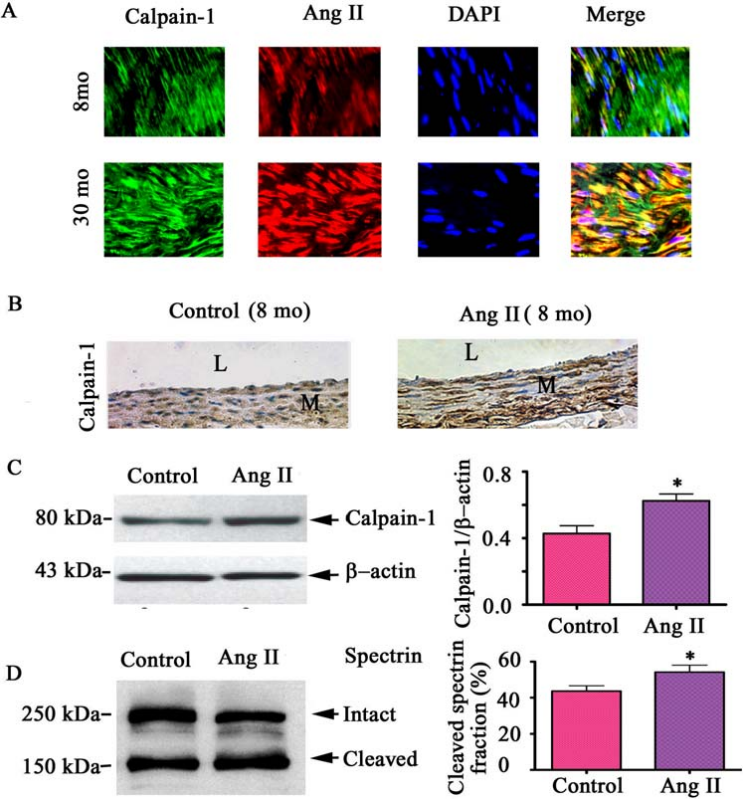
Calpain-1 induction in vivo by Ang II. A. Dual fluorescence labeled calpain-1 (green) and Ang II (red) in the en face medial aortic sections from young (upper panels) and old rats (lower panels). Merged image (right panels, yellow). Nuclei were counterstained with DAPI (blue). Magnification: ×400. B. Photomicrographs (X400) of carotid sections stained for calpain-1 protein (brown color) from young rats infused chronically with Ang II 200 ng/kg/min for 28 days. L = lumen; and M = media. C. Western blots of calpain-1 and β-actin of carotid lysates from young rats infused with saline solution (control) and Ang II (left panel); right panel shows average data. D. Western blots of the calpain substrate, α-II spectrin, of carotid arteries (left panels). Average cleaved spectrin fraction (right panel). *p<0.05, Ang II vs. saline infusion.

Ang II is a known stimulator of calpain-1 expression in cardiomyocytes [32]. Prior studies have shown that Ang II protein abundance increases in the aged aortic wall in vivo and in VSMC in vitro [8], [11], [12]. To determine if Ang II induces calpain activation in the arterial wall with advancing age, we have performed in vivo, ex vivo and in vitro experiments. Double immunolabeling of VSMC showed that increased Ang II is co-localized with increased calpain-1 in VSMC from old aortae (**Fig. 2A** **,** yellow color, right lower panel). We attempted to reproduce features of arterial aging in specimens from young animals. A 28 day infusion of Ang II into young rats (8mo) increases aortic calpain-1 protein staining **(** **Fig. 2B**), protein abundance by 1.5 fold (**Fig. 2C** **),** and activity by 1.2 fold **(** **Fig. 2D** **)** compared to the saline infused (control). Thus calpain-1 activation follows Ang II exposure in young aortae, resembling that of untreated old animals in **Fig. 1** **.**

**Figure 3:**
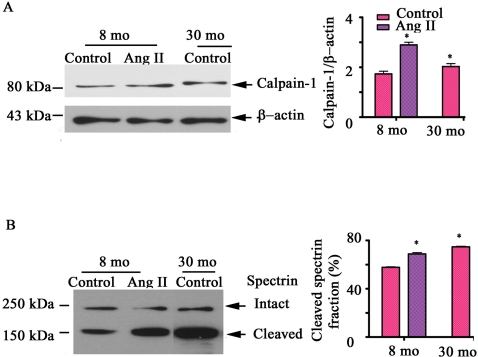
Calpain-1 induced ex vivo by Ang II. A. Western blots of calpain-1 and β-actin of arterial rings from young and old rats treated with Ang II for 72 hrs (left panels); average data (right panel). B. Western blots of the calpain substrate, α-II spectrin, of arterial rings from young and old rats treated with Ang II for 72 hrs (left panel); average data of cleaved α-II spectrin fraction (right panel). *p<0.05, vs. young untreated rings (control).

Ang II infusion *in vivo* alters both circulating cells and hemodynamic factors [11], [34], both of which may affect the expression and activity of arterial calpain-1. To avoid these issues we utilized exposed organoid cultures of aortic rings from young rats, which retain the anatomic relationship among the cells of the vessel, to Ang II (10^−7^ mol/L) for 48 hrs. Calpain-1 protein abundance was increased by 1.7 fold **(** **Fig. 3A** **)** and activity by 1.2 fold **(** **Fig. 3B** **)**, up to the levels of untreated arterial rings from old rats.

**Figure 4:**
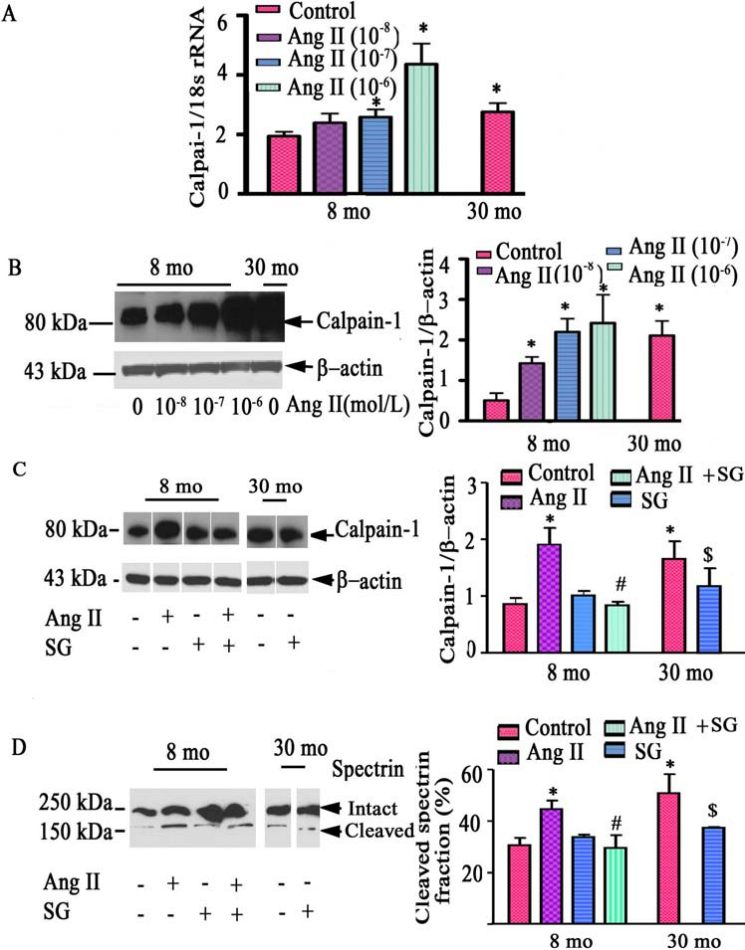
Calpain-1 induction in vitro by Ang II. A. Average calpain-1 transcript abundance from VSMC treated with Ang II for 48 hrs, determined by real time RT-PCR. B. Western blots of calpain-1 of early passage VSMC treated with Ang II for 48 hrs (left panels); average data (right panel). C. Western blots of calpain-1 of early passage VSMC treated with Ang II (10^−7^mol/L) and or SG (10^−7^mol/L), an AT1 antagonist, for 48 hrs (left panels); average data (right panel). D. Western blots of α-II spectrin of early passage VSMC treated with Ang II (10^−7^mol/L) and or SG (10^−7^mol/L), an AT1 antagonist, for 48 hrs (left panels); average data (right panel). *p<0.05, vs. young untreated VSMC (control). #p<0.05, vs. Ang II treated young VSMC. $p<0.05, vs. old untreated VSMC.

To determine whether VSMC in vitro (p3-p5), in fact, retain their properties in vivo, aortic VSMC were isolated from young and old animals. Indeed, calpain-1 transcriptome (**Fig. 4A** **)**, protein expression abundance **(** **Fig. 4B & 4C** **)**, as well as activity (**Fig. 4D** **)** were up-regulated within old VSMC compared to young cells**.** Further, qRT-PCR analysis indicated that Ang II induces young VSMC to increase calpain-1 transcription in a dose-dependent manner, achieving the level of untreated old cells **(** **Fig. 4A** **)**. Likewise, treatment of young cells with Ang II increases calpain-1 protein abundance in a dose-dependent fashion, to the level of untreated old cells **(** **Fig. 4B** **)**. Interestingly, the age- and Ang II-associated increases in calpain-1 protein and activity are substantially reduced by [Sar^1^, Gly^8^]-Angiotensin II acetate hydrate (SG), an AT1 receptor antagonist **(** **Fig. 4C & D** **).**

### Calpain-1 mediates Ang II associated activation of MMP2

**Figure 5:**
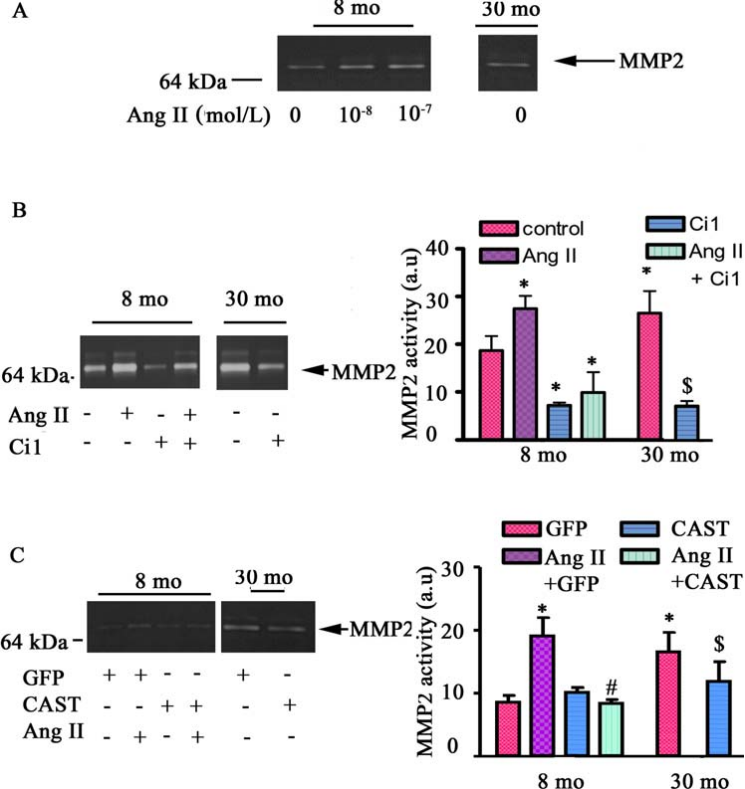
Ang II induces activity of MMP2 mediated by calpain-1 activation. A. A representative PAGE gelatin zymogram from young and old VSMC treated with Ang II for 48 hrs. B. A representative PAGE gelatin zymogram of VSMC treated with Ang II, or Ci 1, a calpain inhibitor, for 48 hrs (left panel); average data (right panel). C. A representative PAGE gelatin zymogram of VSMC infected adenovirus harboring GFP or CAST treated with Ang II (left panels); average data (right panel). *p<0.05, vs. young untreated VSMC (control). #p<0.05, vs. Ang II treated young VSMC. $p<0.05, vs. old untreated VSMC.

We have previously shown that Ang II increases MMP2 activation in the arterial wall in vivo and ex vivo and in isolated VSMC in vitro [11]. Since both calpain-1 and MMP2 are involved in the attachment, spreading, and migration of both fibroblasts and VSMC [8], [11], [23], [24], we sought to demonstrate a link between calpain-1 and MMP2 activation. **Fig. 5A** shows that Ang II increased MMP2 activity in a dose-dependent manner in young VSMC, up to the level of untreated old cells. The increased MMP activity induced by Ang II was abolished by Ci 1, a non-specific calpain inhibitor **(** **Fig. 5B** **).** Additionally MMP2 activity within untreated young and old VSMC was substantially inhibited by Ci 1 **(** **Fig. 5B** **).** To confirm a specific role of calpain-1 in Ang II induced MMP2 activation, young VSMC infected with a virus harboring calpastatin (CAST), an ectopic expression of the endogenous calpain inhibitor, were exposed to Ang II. Figure S1**A** (**Fig S1 A)** demonstrates that CAST over-expresses calpastatin in cultured VSMC. The infection with CAST virus substantially reduced MMP2 activity both in Ang II treated young cells and in old untreated cells (**Fig. 5C**).

### Calpain-1 remodels the cytoskeleton within VSMC

**Figure 6:**
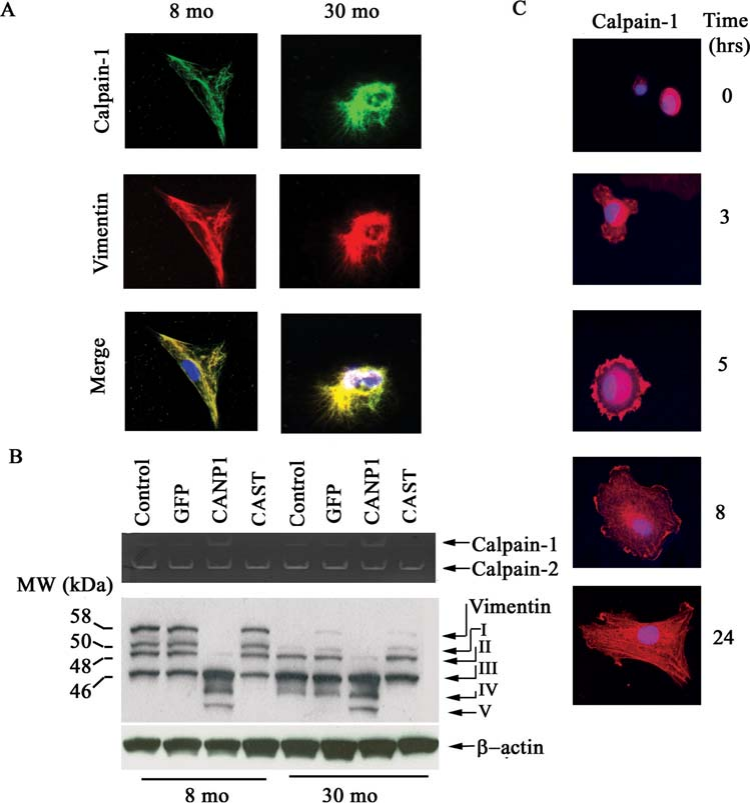
Calpain-1 and vimentin interactions within VSMC. A. Photomicrographs of dual labeling for calpain-1 (green) and vimentin (red) within VSMC from young (left panels) and old rats (right panels); merged image (yellow bottom panels). B. Casein zymogram of VSMC from young and old VSMC infected with a CANP1 or CAST virus (upper panel). Western blots of vimentin from young and old VSMC infected with CANP1 or CAST virus (lower panel). Proteolytic fragments are indicated by arrows and designated I-V. C. Fluorescence labeled calpain-1 protein of VSMC isolated from young rat aortae. VSMC were isolated from the aorta of 8-month-old rat, allowed to adhere on a fibrogen matrix, fixed with methanol and acetone, and stained with calpain-1 antibody (red). The nuclei were counterstained with DAPI (blue). Magnification × 400.

With aging, alterations of VSMC cytoskeletal proteins such as α-SMA and vimentin occur concomitantly with changes in VSMC migration rates [35]. **Fig. 6A** demonstrates that within the old VSMC increased calpain-1 colocalizes with vimentin (**Fig. 6A**, lower right panel, yellow color) and α-SMA (data not shown). Casein zymography (**Fig. 6B**, upper panel) confirms that calpain-1 but not calpain-2 activity in VSMC increase with age. Over-expression of calpastatin did not affect calpain-1 activity in young VSMC, although it decreased calpain-1 activity in old cells. Moreover, calpain-2 activity was not affected by over-expression of calpastatin in either young or old VSMC. These inconsistent effects may be due to different calcium requirements for calpastatin binding to calpain-1 or calpain-2 [36]. Cytosolic calcium concentrations ([Ca ^2+^]) are known to increase with aging [37]. A single vimentin cDNA encodes a 57-kDa intact protein [27]. In addition, we observed intact vimentin protein only in early passage young VSMC, but not in old cells, which showed only small vimentin fragments (**Fig. 6B**). Further, several small fragments are disproportionately distributed in young and old cells: while both young and old cells share fragments II and III, fragment I is more abundant in young cells (**Fig. 6B**, 8 mo control), while fragment IV is unique to old cells (**Fig. 6B**, 30 mo controls).

This characteristic age-associated shift of vimentin fragments suggests that arterial wall calpain-1 may cleave the intact vimentin, generating multiple proteolytic fragments. To test this hypothesis, we also constructed and amplified adenoviruses harboring calpain-1 and over-expressed calpain-1 in VSMC in **Figure S1 B**. Indeed, infection of adenoviruses harboring calpain-1 (CANP1) into young VSMC dramatically increases calpain-1 activity (**Fig. 6B**, **upper panel**) and generates a multiple proteolytic fragment pattern (**Fig. 6B**, **lower panel, 8 mo)**, similar to old uninfected or CANP-1 infected cells (**Fig. 6B**, **lower panel, 30 mo**). Remarkably, infection of old VSMC with an adenovirus harboring CAST indeed inhibits calpain-1 activity (**Fig. 6B**, **upper panel)** and generates a pattern of proteolytic products of vimentin resembling that from the untreated young cells **(** **Fig. 6B**, **lower panel, 30 mo).**

The interactions of calpain-1, α-SMA, spectrin, and vimentin that occur concomitantly with changes in SMC migration with advancing age suggest that the dynamic changes in distribution of calpain associated cytoskeletal proteins may play a role in VSMC attachment and spreading, crucial processes of cell migration. We next determined the spatial and temporal location of endogenous calpain-1 in the attachment processes of VSMC by using time-lapse photomicrography. **Fig. 6C** demonstrates that at the initiation of attachment, calpain-1(red) is abundantly aggregated in the perinuclear region **(0 hr)**, and then begins to protrude outwardly as the cell becomes attached to the substratum **(3 hrs).** At 5 hrs, the cell has become attached, as shown by a completed circular border with calpain-1 preferentially localized at the periphery of the cell**.** At the 8 hr point, the spreading processes have taken place and calpain-1 has continuously expanded peripherally, accompanied by the formation of lammellipodia. Finally, calpain-1 is uniformly distributed once the cell has achieved a fully spread state **(24 hrs).**

### Catalytic activation of calpain-1 is required for age-associated enhancement of VSMC migration

**Figure 7:**
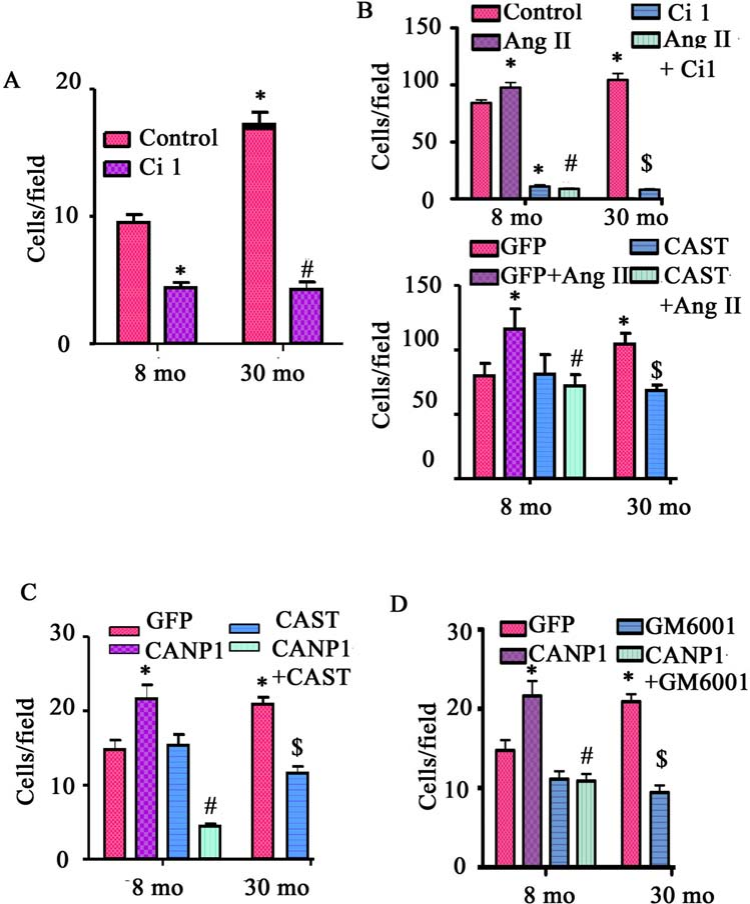
VSMC migration. A. The random migration of VSMC. *p<0.05, vs. young; and #p<0.05, vs. young or old untreated control cells B. The migration of VSMC treated with Ang II or Ci1 (upper panel) or infected with or without a CAST virus (lower panel), in response to a PDGF-BB gradient (10ng/ml) *p<0.05, vs. young untreated VSMC (control). #p<0.05, vs. Ang II treated young VSMC. $p<0.05, vs. old untreated VSMC. C. The random migration of VSMC infected with a CANP1 or CAST virus. *p<0.05, vs. young; and #p<0.05, vs. young infected with CANP1 virus. $p<0.05 vs. old infected with control (GFP) virus. D. The random migration of VSMC infected with CANP1 or GM6001. *p<0.05, vs. young; and #p<0.05, vs. young infected with CANP1 virus. $p<0.05 vs. old infected with control (GFP) virus.

Data presented thus far show calpain-1 within VSMC co-localizes with vimentin (**Fig. 6A** **),** is capable of cleaving spectrin [18], vimentin (**Fig. 6B** **)**, and α-smooth muscle actin (data not shown), and is also linked to Ang II induced activation of MMP2. We addressed the idea that calpain-1 plays a role in MMP2 activation and cytoskeleton restructuring, crucial events relevant to age-associated increased VSMC migration. **Fig. 7A** shows that the random migration capability of early passage VSMC from old rats is increased 1.9 fold compared to that of young VSMC (p<0.05). Interestingly, Ci 1, the exogenous cell–permeable calpain inhibitor, dramatically reduces the random migratory capability of both young and old cells (**Fig. 7A**).

The increased Ang II protein expression within older VSMC is accompanied by an increased gradient of intimal-medial PDGF-BB in the old aorta [11], [38]. PDGF-BB, a potent chemoattractant, enhanced VSMC chemotaxis by 9.1–fold for young (85.4±2.4 vs. 9.1±0.6 cells/field, p<0.05) and by 6.1-fold for old cells (103.6±5.2 vs. 17.2±1.0 cells/field, p<0.05). **Fig. 7B** (upper panel) shows that Ang II treatment increases the migration capacity of young VSMC to the level of untreated old cells. Strikingly, Ci 1 completely abolished the Ang II-/PDGF-BB-induced chemotaxis of both young and old cells. Infection of the adenovirus harboring CAST completely abolished the Ang II-induced VSMC migration increase (**Fig. 7B**, lower panel).

To bypass both Ang II and PDGF-BB signaling, we infected young VSMC with an adenovirus harboring calpain-1 (CANP1) or calpastatin (CAST) cDNA. Over-expression of CANP1 enhanced the migratory ability of young VSMC by 1.5-fold (p<0.05), up to the level of the GFP infected old VSMC **(** **Fig. 7C** **).** Overexpression of calpastatin reduced the migratory capability of old cells, to the level of young cells infected with GFP alone **(** **Fig. 7C** **).** Further, the increased chemotaxis induced by CANP1 infection in both young and old cells was substantially reduced by GM6001, an MMP inhibitor **(** **Fig. 7D** **).** In addition, GM6001 substantially inhibited the migration of old cells **(** **Fig. 7D** **).**

## Discussion

Our study documents multiple novel roles of calpain-1 in age-associated vascular restructuring in rats**.** First, we show that the levels of transcript, protein and activity of calpain-1 within the aged aortic wall and isolated VSMC increase with advancing age. Second, increased Ang II within the aged aortic wall or VSMC [11], per se, mediates the expression and activation of calpain-1. Third, activated calpain-1 is necessary for activation of MMP2 within VSMC, a characteristic feature of the aged aorta. Fourth, increased calpain-1 activity is involved in the exaggerated cleavage of cytoskeletal proteins, including spectrin and vimentin, in old VSMC. Finally, the catalytic activity of calpain is required for the efficient chemotaxis of VSMC induced by Ang II or PDGF-BB.

Our results show that increased Ang II within the aged aortic wall or VSMC stimulates the expression and activation of calpain-1 in vivo, *ex vivo* and *in vitro*. Chronic infusion of Ang II into young rats for 28 days enhances calpain-1 protein abundance and activation in the arterial wall, similar to that of the old animal. Ang II infusion, in vivo, effects changes in multiple factors, i.e. circulating cells and hemodynamics [11], [34], each of which impacts on the expression and activation of calpain-1 [39]–[41]. We employed an organoid culture of arterial rings to show that as anticipated, Ang II treatment, per se, in young VSMC promotes calpain-1 expression and activation, reaching the levels of rings from old untreated rats. Further, exposure of isolated VSMC from young rats to Ang II increases the activity of calpain-1 to the levels of untreated cells. These effects are substantially inhibited by SG, an AT1 receptor antagonist. The present results are consistent with previous reports in other cells or organs that support the idea that AT1 signaling exerts a causal role in calpain-1 activation. [32], [42], [43]. Factors present in the aged arterial wall facilitate the transcriptional and post-translational modification of calpain-1 that leads to its activation.

The transcriptional activation of calpain involves the promoter/enhancer motif, 12-*o-*tetradecanoyl-phorbol-13-acetate response element (TRE), which binds AP-1 [28], [44], a dimer consisting of c-fos and c-jun [45]. AP-1 activity in the nuclei of VSMC which is directly or indirectly triggered by Ang II, and its associated signaling molecules ET-1, epinephrine, and ROS [8]–[15], [17], [46], [47], likely switches on calpain-1 gene transcription [28], [46], [48], [49]. Further, the age-associated increase of these vasoactive factors within the arterial wall open L-and T-type calcium channels, increasing Ca^2+^ influx or redistributing intracellular stored Ca^2+^, contributing to a transient increase in cytosolic Ca^2+^ [50], [51], which provides a niche for facilitating the activation of calpain-1 following its translation.

Increased MMP2 activity, due in part to age-associated increases in aortic wall MT1MMP and reductions of TIMP2, is a characteristic feature of the aged arterial wall [12]. The present findings suggest that calpain-1 activation is a most likely event within the Ang II/MMP2 signal axis during arterial aging because calpain-1 activation is necessary for Ang II induced activation of MMP2 within VSMC. When young VSMC are exposed to Ang II, MMP2 activation increases in a dose-dependent manner, up to the level of old untreated cells. Importantly, increased MMP2 activity is completely abolished by Ci 1, an inhibitor of calpain. Further, Ci 1 treatment also substantially reduces MMP2 activation in both young and old untreated VSMC**.** These findings are endorsed by previous reports in fibroblasts and leukemic cells [23], [24]. Neither MMP2 mRNA, nor MMP2 activity can be detected in a calpain-1 null mouse embryonic fibroblast transformed by SV40 large T-antigen (TAg-capn 4 -/-mEF) [23]. However, in TAg-capn4-/-mEF, a low level of secreted MMP2 is restored by transfection with a vector expressing rat calpain, which partially restores calpain activity [23]. Interestingly, the calpastatin-derived calpain inhibitor, CP1B, reduces mRNA expression and secretion of MMP2 in the leukemic cell line THP-1[24]. In addition, we also demonstrate that an infection of VSMC with CAPN1 increases the transcription and protein expression of MT1-MMP, an activator of MMP2, and decreases protein levels of TIMP2, an inhibitor of MMP2 (Jiang LQ, unpublished data). Thus, calpain-1 activation has a role in creating an imbalance of MMP2 activators and inhibitors that contribute to increased MMP2 activity within VSMC.

VSMC exhibit a striking plasticity and are able to change from a differentiated contractile phenotype to a more immature, synthetic phenotype with aging [2], [3]. This includes a prominent cytoskeletal reorganization and construction of a large secretory apparatus to produce Ang II, MMP2, MCP-1, and latent TGF-β1 [11], [12], [16], [31]. As a result, the aged cells lose their contractility and are able to migrate, proliferate, and secrete extracellular matrix components. In vivo, this VSMC phenotypic shift is a crucial event in the remodeling of the arterial wall with aging. The increased calpain-1 is involved in the restructuring of the cytoskeleton, which includes actin, spectrin, and vimentin, within VSMC with aging [35]. Our results support the idea that the catalytic activity of calpain is an unequivocal critical regulator of efficient VSMC chemotaxis. We show that cytoskeletal proteins are substrates of natural or ectopically expressed endogenous calpain-1 within VSMC, and that calcium-dependent vimentin cleavage was substantially inhibited by the ectopic over-expression of a calpain inhibitor, calpastatin. The strikingly different pattern of multiple fragments of vimentin in young and old VSMC in this present study reflect the importance of cytoskeleton remodeling with age. Since the cytoskeleton plays a crucial role in the attachment, spreading, and migration of cells [18], [19], [22], [24], [26], [27], [52], it is reasonable that age-associated changes in calpain are involved in age differences in the attachment and spreading of VSMC.

In the present study, we further confirm that VSMC migratory capability increases with aging [8], [9], [11], [16], [35]. This age-associated VSMC migration increase is exaggerated by Ang II treatment, over-expression of calpain-1, and PDGF-BB chemoattractant. The catalytic activity of calpain is required for efficient chemotaxis of VSMC induced by Ang II or PDGF-BB. These effects are abrogated by GM6001, an MMP2 inhibitor, or calpain-1 inhibitor, Ci 1, or over-expression of calpastatin. Notably, Ci l unselectively reduces the migration capacity of VSMC from both young and old animals. These results are consistent with reports on fibroblast and THP-1 cells [23], [24]: interruption of calpain activation can impair cell movement, as shown in calpain-deficient mouse fibroblasts and CHO cells, while over-expression of calpastatin or exogenous calpain inhibitor impedes cell spreading and migration.

In summary, our results demonstrate an age-associated increase of calpain-1 gene transcripts, protein levels and activity in the old aortic wall; increased Ang II induces calpain-1 expression in the aortic walls in vivo and ex vivo and VSMC in vitro; the Ang II mediated age-associated increased MMP2 activity, is blocked by calpain inhibitor 1 or CAST, indicating that calpain activity is required for MMP activation by Ang II treatment. An increase in old VSMC migratory capacity is mimicked in young VSMC by over-expression of calpain-1. Collectively, these findings point to increased calpain-1 activity, as a central component of the exaggerated Ang II signaling pathway, which is involved in age-associated arterial remodeling. Thus, targeting calpain-1 is a potential approach to delay or reverse the age-associated arterial remodeling that is also associated with diseases such as hypertension, atherosclerosis, and stroke.

## Materials and Methods

### Animals and Ang II infusion

8-month (young, n = 40) and 30-month (old, n = 20) Fisher 344 X Brown Norway (F344XBN) male rats were obtained from the National Institute on Aging contract colonies (Harlan Sprague-Dawley, Indianapolis). Twenty young rats were randomly allocated into 2 groups, and implanted subcutaneously with osmotic minipumps (Model 2004, Alzet Corp) in the midscapular region, according to methods described previously [11]. Briefly, rats were infused with Ang II at a rate of 200 ng/kg/min (Ang II infusion, n = 10) and rats infused with saline served as untreated young controls (control, n = 10). The animal protocol was approved by the Institutional Animal Care and Use Committee and complied with the guide for the care and use of laboratory animals (National Institutes of Health publication No. 3040-2, revised 1999).

All of the animals were sacrificed by an overdose of sodium pentobarbital, and thoracic aortae were immediately removed, isolated, and processed as described previously [11]. In addition, a 3–4 mm in length segment of thoracic aorta from young and old rats (n = 4 per group) was explanted. This segment was cut longitudinally, flattened on a wooden board, and embedded in paraffin for en face observation by immunofluorescence.

### Organ Culture

Aortic rings were explanted and cultured as described previously [11], [31]. Briefly, after dissection of adventitial tissue, aortic segments were placed in 6-well dishes containing DMEM+F12 (1∶1) (GIBCO BRL) and antibiotics (penicillin 100 U/mL, streptomycin 100 mg/mL) and supplemented with transferrin 5 µg/ml, and insulin 5 µg/mL. The vessel segments were then maintained in a tissue culture incubator at 37°C and exposed to various experimental conditions for the times indicated. The vascular segments were frozen in liquid nitrogen and the supernatants were stored at −80°C until use.

### Generation of recombinant adenoviruses

The human calpain-1 full-length cDNA fragment was obtained as described previously [39]. The calpain-1 cDNA (2210 bp) was amplified by polymerase chain reaction (PCR) with primers containing Xho I and Hind III restriction sites at the 5′ and 3′ ends, respectively. The upstream primer is 5′-CTCGAGCACCATGTCGGAGGAGATC-3′ and the downstream primer is 5′-AAGCTTGGCAGACGAGGAGGGAGG-3′. The rat calpastatin full-length cDNAs were amplified by reverse transcription-PCR (RT-PCR) from 2 µg of total RNA extracted from rat aorta. The primers for the calpastatin cDNA fragment (2003 bp) which added Kpn I and Xba I restriction sites at the 5′ and 3′ ends respectively were as follows: 5′-GGTACCTCTCCCAGAATGAG-3′ 5′- TCTAGATACCAAAATGCATAAAC-3′. The PCR fragments were ligated to the pGEM-T vector and sequenced. The calpain-1 and calpastatin were then cut by Xho I and Hind III or Kpn I and Xba I, respectively and then ligated to pAdTrack-CMV which has a reporter gene expressing green fluorescent protein. The adenoviral backbone plasmid pAdEasy-1 and shuttle plasmid pAdTrack-CMV were a gift from Dr Bert Vogelstein (John Hopkins Oncology Center Baltimore Maryland, USA). The replication-defective recombinant adenovirus encoded calpain-1 (CANP1) or calpastatin (CAST) together with green fluorescent protein were constructed by homologous recombination, as described in a previous study [53]. An adenoviral vector expressing green fluorescent protein (GFP) served as a control virus. Standard viral amplification and cesium chloride purification methods were used to amplify and purify these adenoviruses. The titer for each adenovirus in HEK293A cells was determined via a dilution assay.

### VSMC Isolation, culture, and infection

Vascular smooth muscle cells were enzymatically isolated and cultured as previously described [16]. Briefly, F344XBN rat thoracic aortas were rinsed in Hanks balanced salt solution (HBSS) containing 50 µg/mL penicillin, 50 µg/mL streptomycin and 0.25 µg/mL amphotericin B (Gibco). After digestion for 30 min in 2 mg/mL collagenase I solution (Worthington Biomedical, Freehold, New Jersey) at 37°C, the adventitia and intima were removed from the vessel media layer, which was placed overnight in complete medium (DMEM plus 10% FCS). On day 2 the vascular media was further digested with 2 mg/mL collagenase II/0.5 mg/mL elastase (Sigma) for 1 hour at 37°C, and the isolated cells were washed and plated in complete medium. In all cases, >95% of cells stained positive for α-smooth muscle actin (α-SMA).

Early passage (p3-p5) VSMC were untreated, or pre-treated with Ang II (100 nmol/L) only or together with [Sar^1^, Gly^8^]-Angiotensin II acetate hydrate (SG, Cat# A-7401, Sigma, St. Louis, MO) (100 nmol/L), an AT1 blocker, or together with the calpain inhibitor, Ci 1 (100 µmol/L, Cat# 208719, Calbiochem, San Diego, CA) for 48 hours in 0.1% FBS for Western blot and Polyacrylamide Gel Electrophoresis (PAGE) gelatin zymographic analysis.

Adenoviral infection of VSMC was performed with a multiplicity of infections (MOI) of 100. After forty-eight hours of adenoviral infection, total cellular proteins were isolated for Western blot or migration assays.

### Real-time PCR

Quantitative Real-time PCR (q-PCR) was performed using the SYBRGreen PCR-based protocol in a 384-well plate format (Applied Biosystems) as described previously [16]. All the primers used for Real Time PCR analysis have been designated using Primer Express software 1.5 (Applied Biosystems, Foster City, CA), and synthesized by Invitrogen Life Technologies (Carlsbad, CA). The primer sequence sizes are listed in **Table 1**.

**Table 1:** Primers sequences and amplified fragments size used for real-time PCR

	Upstream primer sequence	Downstream primer sequence	Fragment size (bp)
Calpain-1	CCGGGACTTCATACGTGAGT	AGGTGCCCTCGTAAAATGTG	110
18s rRNA	CGCGGTTCTATTTTGTTGGTTT	GCGCCGGTCCAAGAATTT	103

RNA was extracted from frozen aortae or cultured VSMC using Trizol reagent. RNA (2 µg) was reverse transcribed for 30 minutes at 48°C using random hexonucleotides according to the manufacturer's instructions (Applied Biosystems Foster City CA). Real-time PCR was performed according to the SYBRGreen PCR protocol (Applied Biosystems Foster City CA). Each sample has been tested in quadruplicate. The reaction conditions were: 10 min at 95°C (one cycle); 30 sec at 95°C; 30 sec at 60°C; and 30 sec at 72°C (40 cycles). Gene-specific PCR products were continuously measured by an ABI PRISM 7900 HT Sequence Detection System (PE Applied Biosystem Norwalk, CT). The PCR product sizes were verified by agarose gel electrophoresis. Samples were normalized to the expression of the “housekeeping” gene, the 18S rRNA, which did not change with age. Data are expressed using the formula: quantity = 10^−(Ct-Y intersept)/slope value)^ where Ct represents the threshold cycle value.

### Immunostaining

Immunohistochemistry and immunofluorescence were performed according to the methods described previously [8], [11], [12], [16]. The detailed primary antibodies used for immunostaining are listed in **Table 2**.

**Table 2:** Primary antibodies used for immunostaining (IS) and Western blotting (WB)

Antibody	Species	Titer for IS	Titer for WB	Source
Calpain-1	Rabbit	1∶100	1∶2500	Sigma, St. Louis, MO
α-II spectrin	Mouse		1∶3000	BioMol, Plymouth Meeting, PA
α-SMA	Mouse	1∶80		DAKO, Glostrup. Denmark
Ang II	Rabbit	1∶50		Peninsula Laboratories, Inc., Dan Carlos, CA

### Western Blot Analysis

Western blotting of supernatants from thoracic aortae homogenates or VSMC lysate products was performed according to the methods described previously [11], [12], [16]. Primary antibodies for the Western blotting are listed in **Table 2** **.**

### Calpain and MMP2 activity assays

The calpain activity in rat aorte or cultured VSMC was determined via a measurement of the fraction of calpain-specific spectrin cleaved products (SBDP), as described previously [54]. Briefly, lysates from rat aortae or cultured VSMC were immunoblotted with an antibody against *α*-II-spectrin. This antibody recognizes intact 280-kD *α*-II-spectrin and calpain-specific cleaved 150 and 145-kDa fragments. The spectrin fraction is calculated by dividing the cleaved spectrin densitometry by total spectrin densitometry. The total spectrin densitometry is equal to the intact densitometry plus the cleaved densitometry.

In addition, casein zymography of VSMC was performed according to the modified protocol described previously [55]. Cultured VSMCs were collected with lysis buffer containing 50 mM HEPES, 159 mM NaCl, 10%Glycerol, 1%Triton X-100, 5 mM EDTA, 15 mM β-mercaptoethanol, 1 mM PMSF, 2 µg/mL Leupeptin, into a sterile tube. Cells were homogenized by vortexing for 30 sec and centrifuging for 15 min at 4°C at 13,500 rpm. The supernatant was used fresh or stored at −80°C until use. Casein (0.2%, w/v) was copolymerized with 10% acrylamide (w/v), HI buffer (25 mM HEPES, 20 mM imidazole, pH 7.4), 20 mM EGTA as a separation gel, 5% acrylamide (w/v), HI buffer (no casein) as a stacking gel were poured into minigel casts (Bio-Rad). Ammonium persulfate (0.04%, w/v) and TEMED (0.028%, v/v) were used to catalyze the polymerization. 20 µg of cell lysate were mixed with 2× sample buffer (0.1 M Tris-HCl buffer, 10 mM EDTA, 20% glycerol, 0.2% bromophenol blue, and 0.3 M β-mercaptoethanol). The casein gels were pre-run at 125V with a buffer containing 40 mM HEPES, 30 mM imidazole, 0.625 mM EDTA and 15 mM β-mercaptoethanol, pH 7.4 for 30 min at 4°C. Samples were then loaded into the wells and run for another 3 hrs at 4°C at 125V. The gel was then removed and incubated in developing buffer containing 25 mM Tris, 25 mM MOPS, 20 mM imidazole, 18 mM HEPES, 6.7 mM CaCl_2_, and 15 mM β-mercaptoethanol with slow shaking for 60 min(with 2 changes of buffer). The gel was then further incubated overnight (16–20 hr) at 37°C in the same developing buffer. Finally, the gel was stained with Coomassie blue.

MMP2 activity assays were determined via PAGE gelatin zymography according to the methods described previously [8], [11], [12].

### VSMC Migration

The following VSMC Migration was performed according to the methods described previously [56]. Briefly, modified Boyden chambers were equipped with 8 µm pore-size polycarbonate filters (Neuroprobe, Gaithursburg, MD) coated with Matrigel (BD Biosciences, Palo Alto, CA). VSMC were untreated, or pre-treated with Ang II (100 nmol/L) only or together with Ci 1, an inhibitor of calpain (10 µmol/L ); VSMC were infected with adenovirus harboring calpain-1 (CANP1) or calpatatin (CAST) cDNA; or infected VSMC were treated with or without GM6001 (15 µmol/L) for 24 hours in 0.1% FBS. Two hundred and twenty µL of migration medium (MM, DMEM with 0.1% BSA) was added to the lower chamber with or without platelet-derived growth factor (PDGF-BB, 10 ng/ml) for chemoattract and random migration assay, respectively. VSMC (10^6^/mL) were placed in the upper chamber in 200 µL of MM, performed in triplicate. The assay was stopped after 4 hours at 37°C, and cells that had crossed the basement membrane and migrated to the lower side of the filter, were fixed and stained using the HEMA3 system (Curtin Matheson Scientific, Inc., Houston, TX). Four random fields were counted at 400× magnification for each filter.

### Statistical Analysis

All results are expressed as the mean±SEM from at least three independent experiments. Statistical comparisons for multiple group differences were made via an ANOVA, followed by Bonferroni post hoc tests. Two-age group differences were determined via Student's t- tests. A p value of <0.05 was taken as statistical significance.

## Supporting Information

Figure S1 Over-expression of calpain-1 or calpastatin by adenovirus infection. A. Fluorescence photomicrographs show that Adenovirus (GFP or CAST) infected into VSMC. GFP expression was visualized after 48 hrs. Western blots of calpastatin from VSMC infected with CAST virus (right panel). B. Adenovirus (GFP or CANP1) infected into VSMC. GFP expression was visualized by fluorescence microscopy after 48 hrs. Western blots of calpain-1 from VSMC infected with CANP1 virus (right panel).
(1.24 MB TIF)
