# Supplementary figures and images for: Increased Aortic Calpain-1 Activity Mediates Age-Associated Angiotensin II Signaling of Vascular Smooth Muscle Cells
Source: PLoS One. 2008 May 21;3(5):e2231. doi: 10.1371/journal.pone.0002231 (PMC2373882; doi:10.1371/journal.pone.0002231)

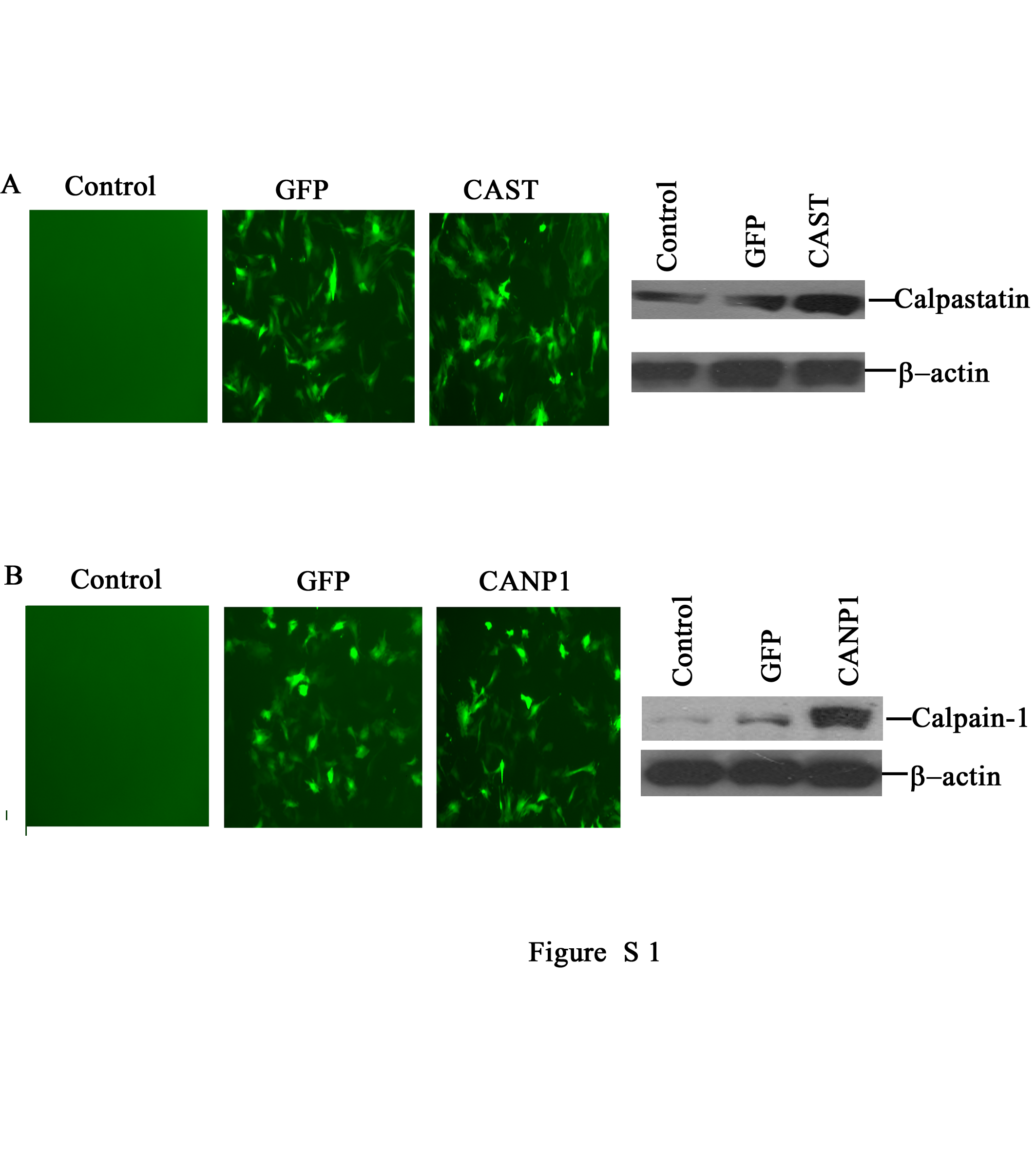

Supplement: Figure S1 — Over-expression of calpain-1 or calpastatin by adenovirus infection. A. Fluorescence photomicrographs show that Adenovirus (GFP or CAST) infected into VSMC. GFP expression was visualized after 48 hrs. Western blots of calpastatin from VSMC infected with CAST virus (right panel). B. Adenovirus (GFP or CANP1) infected into VSMC. GFP expression was visualized by fluorescence microscopy after 48 hrs. Western blots of calpain-1 from VSMC infected with CANP1 virus (right panel). (1.24 MB TIF) [file pone.0002231.s001.tif]
